# From pain gaslighting to gender biases in women’s accounts of hysteroscopy: A qualitative reflexive thematic analysis

**DOI:** 10.1177/17455057261440884

**Published:** 2026-04-20

**Authors:** Susanne K. Cromme, Richard Harrison, Katherine A. Finlay

**Affiliations:** 1 School of Psychology and Clinical Language Sciences, University of Reading, UK

**Keywords:** hysteroscopy, gynaecology, qualitative, gender pain gap, women’s health

## Abstract

**Background:**

Despite being regarded as the gold standard, outpatient hysteroscopy (OPH) is associated with inconsistent outcomes and pain, while the clinical, organisational, and personal determinants shaping patient-centred experience remain poorly characterised.

**Objectives:**

This study aimed to harness the authenticity and richness of naturally occurring online qualitative data to explore the clinical, organisational, and personal factors that shape women’s hysteroscopy experiences, offering vital insights for service improvement.

**Design:**

An in-depth qualitative investigation of hysteroscopy experiences, as shared by individuals on a publicly accessible online discussion forum.

**Methods:**

Four thousand seven hundred sixty-nine posts across 277 discussion threads published between 2018 and 2024 were collected from Mumsnet.com, representing 1971 forum users discussing their personal hysteroscopy experiences. Posts were analysed using reflexive thematic analysis, informed by a constructivist epistemology and a latent, inductive analytic orientation, to capture both the depth and diversity of online contributions.

**Results:**

Five themes captured women’s specific hysteroscopy experiences: (1) Contingent Consent, (2) Unacknowledged Vulnerability, (3) Analgesia Roulette, (4) Gynaecological Pain Gaslighting, and (5) Gendered Pain Gap. These themes delineate a hysteroscopy pathway where consent is shaped by limited choices and misinformation, vulnerability is heightened by procedural exposure, pain relief is inconsistently applied, women's suffering is routinely dismissed, and gender biases reinforce unequal standards of care.

**Conclusion:**

This study identifies clinical blind-spots that contribute to perceptions of systemic neglect in women’s gynaecological health care, evidenced by inconsistent pain management, inadequate consent, and gendered biases in OPH. These findings present an opportunity to inform structural reforms that advance equitable, patient-centred gynaecological care and improve clinical accountability.

## Plain language summary

Women’s experiences of hysteroscopy in the UK

Plain Language Summary: A hysteroscopy is a common medical procedure that allows doctors to look inside the womb and is often the first step in investigating symptoms such as heavy or irregular periods. In the UK, it is usually carried out in an outpatient clinic, meaning women are awake during the procedure. While hysteroscopy is considered the “gold standard” test to identify problems inside the womb, women report very different experiences. Some find the procedure tolerable, while others describe severe pain and distress. This study explored women’s experiences of outpatient hysteroscopy by analysing unprompted, real-life conversations shared on Mumsnet, a popular online discussion forum. We collected 4,769 posts written by 1,971 women between 2018 and 2024 and analysed them to identify common themes. Five key issues emerged:

• Consent is shaped by limited choice: Many women felt they were not fully informed about what to expect or about alternatives to the procedure.

• Women feel vulnerable: The intimate nature of the procedure and the clinical environment left many women feeling exposed and anxious.

• Pain relief is inconsistent: Some women were offered effective pain management, while others were left to cope without it.

• Women’s pain is dismissed: Reports of extreme pain were sometimes minimised or ignored by healthcare professionals.

• Healthcare inequalities: Women felt their pain was taken less seriously than pain experienced by men or pain in other areas of health care.

These findings suggest that many problems with hysteroscopy are not only related to the procedure itself but also how services are organised, information is communicated, and concerns are responded to. Improving these areas could make hysteroscopy a safer, more equitable, and more supportive experience. Our research highlights the need for healthcare providers and policymakers to ensure that women’s voices shape gynaecological care so that experiences are taken seriously and services better reflect women’s needs.

## Introduction

Diagnostic gynaecological procedures present a significant challenge for many women worldwide. Approximately 1-in-3 women live with heavy menstrual bleeding, 1-in-10 women experience endometriosis, and 2-in-3 women have a lifetime risk of uterine fibroids,^1^ all requiring hysteroscopy as their first line of treatment.^2^ In the United Kingdom, hysteroscopy is a procedure predominantly performed in the outpatient setting,^3^ with 71,000 procedures undertaken per year in England.^4^ Hysteroscopy numbers are rising: From March 2018, NICE recommended hysteroscopy as the initial intervention for heavy menstrual bleeding (HMB)^5^ leading to an additional 10,000 hysteroscopies per year in England alone.^6^ Though hysteroscopy is considered the gold standard for visualising the uterine cavity,^7,8^ procedural success rates vary significantly, ranging from 77% to 97.2%.^9–12^ Failure to complete outpatient hysteroscopy (OPH) is predominantly linked to procedural pain^10,13–15^: More than 85% of patients experience pain, with as many as 15%–34.8% of women reporting severe pain during OPH^12,16,17^ and only 7.8% of patients reporting no pain at all.^16^ While pain and patient satisfaction reports^7,16,18,19^ provide some insight into the experience of hysteroscopy, such reports have been criticised as presenting limited, unidimensional representations of the true impact of the procedure.^7,16,18,19^ The lived experience of hysteroscopy care is critically under-researched,^20–22^ restricting the ability to apply patient experience into clinical improvement.

The need to assimilate patient perspectives into clinical care through qualitative research is increasingly recognised as an important adjunct to improving the delivery of hysteroscopy services^17,21^ and is recommended as a priority by the Royal College of Obstetricians and Gynaecologists (RCOG).^23^ Women use online discussion forums to seek advice, share experiences, and access support from others in similar situations,^24–29^ providing a source of rich qualitative data. This study aimed to explore the lived experience of hysteroscopy in the United Kingdom, as represented on Mumsnet.com, seeking to identify the primary concerns of patients, with the potential to optimise clinical care.

## Methods

Ethics approval and consent to participate: Ethics approval was obtained from University of Reading School of Psychology Ethics Research Committee approval (2023-096-KF). The study analysed posts from a publicly accessible online forum viewable without registration; in line with guidance on internet-mediated research,^30^ individual consent was not required. No interaction with users occurred, and all quotations were anonymised to minimise potential harm. This research is reported according to the consolidated criteria for reporting qualitative research (COREQ).^31^ See Supplemental Material.

## Design

This study utilised a qualitative, reflexive thematic analysis of social media posts. Mumsnet was identified as a particularly purposeful source meeting the criteria of UK-wide reach, large and active female userbase, and frequent publicly accessible discussions on women’s health. Mumsnet.com is a UK-based parenting site with 33.1 million monthly visits and 700,000 posts per month. It attracts a diverse user base, with 62.2% aged 25–54 and 52.61% identifying as female.^32,33^ Mumsnet has been utilised by researchers across a range of disciplines as a repository of women’s perspectives and experiences, encompassing contemporary political debates,^34,35^ socio-cultural dynamics,^36–38^ and health-related issues.^39–41^ Importantly, accounts shared on Mumsnet capture experiences from across the United Kingdom, thereby overcoming the geographical and organisational limitations of studies restricted to single NHS trusts. Forum posts and replies available on Mumsnet’s public discussion boards emerge naturally without researcher prompting or alignment with a pre-existing theoretical framework.^42^ In line with established guidance for internet-mediated research,^43^ the forum was treated as a public space. The authors recognise that contributors did not post for the specific purpose of research, but as a publicly available record of their thoughts. To respect this, no interaction with users occurred, no attempts were made to identify individuals, and all quotations were anonymised to uphold ethical standards.^44^

## Data generation

**Figure 1.**
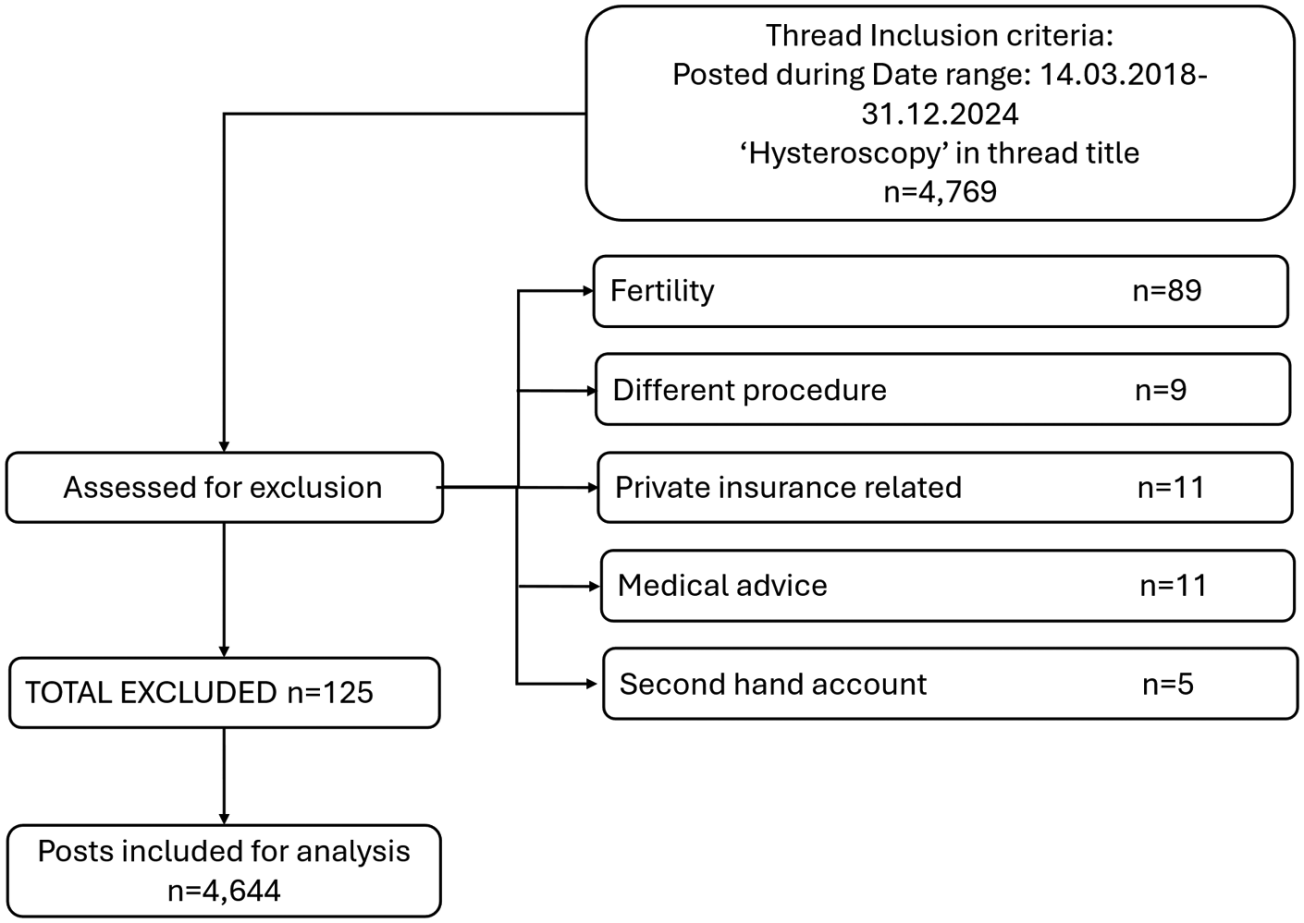
Inclusion flowchart.

Data were generated from Mumsnet representing a purposive sample. The data extraction timeline began from the point of adoption of NICE guideline no. NG88,^5^ on OPH, 4 March 2018 to 31 December 2024 inclusive. Posts that described personal experiences of hysteroscopy were included for analysis; posts relating to fertility, other procedures, medical advice-seeking, second-hand accounts, or the costs of private insurance were excluded (Figure 1). Threads titled with the keyword “Hysteroscopy” were extracted using R Statistical Software (V4.1.2)^45^ using packages rvest^46^, dplyr^47^, and tidyr.^48^ Mumsnet is an anonymous portal with self-selected pseudonyms, which means that demographic data of users are unavailable. For purposes of anonymity, usernames have been omitted.

## Data preparation

Two hundred seventy-seven discussion threads, containing 4769 posts from 1791 individual forum users, were scraped by SKC from the forums. Posts that contained second-hand accounts, medical advice, and off-topic discussions (fertility procedures, private insurance, and private healthcare pricing) were removed to ensure data integrity and relevance to the research question.^49^ After exclusions, 261 threads and 4644 posts remained (Figure 1). Typographical errors were amended to aid readability.^50^ Posts were analysed in MAXQDA.^51^

## Data analysis

Forums often contain extensive, detailed discussions that include multiple perspectives on a topic.^52^ Reflexive thematic analysis^53^ is designed to capture both the diversity and depth of these contributions and to focus on the coherence of the analysis, not on reaching a point of data saturation^54,55^ as would have been intended by the positivist paradigm. The analysis maintained a constructivist epistemological stance that recognises meaning as co-constructed between participants, researchers, and context.^56^ All posts were reviewed multiple times following a latent inductive orientation to the six Steps of RTA^53^: The analysis was led by SKC, an integrative psychotherapist (female, MSc), during familiarisation. Preliminary codes were then created and reviewed, with the coding scheme discussed and adjusted by SKC and KAF (chartered health psychologist/associate professor, female, CPsychol, PhD) and RH (women’s health researcher/lecturer, male, PhD). None of the authors have personal experience of hysteroscopy. The study was designed to generate naturalistic insights from online discussions rather than to examine clinically nuanced decision-making and therefore did not include a clinician as part of the research team. All members of the research team primarily work in clinical research pertaining to women’s health. Themes were created following open discussion and development of consensus between authors about the codes and their clustering.^57^ In line with standardised recommendations,^53^ divergent interpretations were explored within the research team through structured reflexive dialogue, returning to the original data extracts, and deep consideration of how theoretical assumptions and researcher positionality shaped interpretation. Themes were then further developed, critically reviewed, and reshaped collaboratively before finalising theme names and writing the reports. A reflective journal was kept throughout all stages of analysis and analytic decisions were documented as part of an audit trail in Microsoft Excel, enabling transparency in how codes were refined, collapsed, or reconfigured during theme development.

## Analytical transparency and reflexivity

To address sample representativeness and bias, Mumsnet forum posts were conceptualised for the purpose of this study as a large archive of naturally occurring UK health narratives rather than as a direct proxy for the wider OPH population, consistent with established approaches to online qualitative research.^58–60^ As such, we acknowledge self-selection and differential posting, whereby individuals with particularly salient or distressing experiences may be more motivated to contribute, potentially amplifying negative accounts.^61^ A systematic and exhaustive site search was undertaken, and all posts containing the term “hysteroscopy” within the defined timeframe were included without discretionary or directional sampling. Findings were interpreted as contextualised accounts of experience and sense-making within this online forum rather than estimates of prevalence or typicality.

## Results

Five themes were constructed representing the specific experiences of women along the hysteroscopy pathway: (1) Contingent Consent, (2) Unacknowledged Vulnerability, (3) Analgesia Roulette, (4) Gynaecological Pain Gaslighting, and (5) Gendered Pain Gap (Table 1).

**Table 1.** Themes developed during reflexive thematic analysis.

Theme	Quote	ID
Contingent consent	*“Stopping halfway through the procedure is touted here and made to sound easy. When a room full of people are invested in just getting it done, it is clear they are on a tight schedule, you also just want it over with, etc. There are not many women who would halt at that juncture.”*	172
	*“As part of the consent process there should be a mention of alternatives with risk and benefits explained [. . .] and yet I haven’t been through any consent process. I have had a letter confirming my appointment date for an outpatient hysteroscopy with no discussion whatsoever about the alternatives. I wonder how many other women have the same experience which completely contradicts the British Society for Gynae Endoscopy’s statement.”*	4
Underacknowledged vulnerability	*“[a] seemingly unsterile procedure with some random woman doing a bad job of chaperoning, walking in and out the room while my bits were exposed to the elements.”*	767
	*“It’s [expletive] barbaric the way women are treated. The only way we can protect ourselves is to keep ourselves fully informed and stand up to the bullies working in medicine. But what about more vulnerable women? Or younger less experienced women? Who is standing up for and protecting them?”*	85
Pain roulette	*“Some hospitals do hysteroscopies under GA as standard, and others seem reluctant to even give pain relief at all, and of course there’s a range of policies between them.”*	114
	*“The kicker is that no-one knows where they will fall on this line [pain experience] until the procedure is happening; there is no other way to find out! So why take the chance?”*	128
Gynaecological pain gaslighting	*“I was told by the consultant, in quite an irritable way, that if I couldn’t cope with the ‘discomfort’, I’d have to have it done under a GA. Like I was making a fuss over something so minor, it was embarrassing for him to witness it. This is awful, dismissing serious pain as ‘discomfort’. Horribly dismissive way to treat someone.”*	402
	*“I was then wheeled into a full-on operating theatre, heaps of people, bright lights, etc. It seriously was the most horrendous pain I’ve ever experienced. Gas and air, no relief. I begged him to stop, absolutely bawling, not being able to breathe. Was told it’s because I had had C-sections. Making it sound like it was my fault. He marched off, a nurse helped me back to my curtained off bed, I fainted, nearly vomited everywhere. Was left there to put my pants back on and make my way home. My husband picked me up, had no idea what was happening until I called him sobbing my guts out. I tried to make a complaint to my female GP, who was dismissive. I was booked in 2 weeks later with a general. I was sick, shocked, shaken, even typing this is making me feel wobbly.”*	225
Gendered health care	*“I genuinely don’t believe any other speciality would treat a patient in that manner, there’s no way they’d give a man extensive surgery to his genitals and then tell him to get up and get on with it, it wouldn’t happen. I worked on a surgical ward for years – different world to gynae.”*	368
	*“Absolutely agree that female pain and distress from invasive gynaecological procedures is a feminist issue. The way that women are treated as less than fully sentient beings with autonomy and a right to information raises massive issues for patients and professionals and contributes to other problems and keeping women away from healthcare after these awful experiences. And the psychological impact it has on us before, during and afterwards seems to be ignored. It’s misogyny.”*	705

## Theme: contingent consent

Consent provided by women depended on their understanding that the procedural information offered about hysteroscopy was accurate and that consent could be withdrawn. Inadequate patient information undermined their ability to make informed choices, triggering feelings of hurt and disappointment:

I have since [the hysteroscopy procedure] found out that there was at least one other option available to me that wasn’t even discussed, but which I think I would have preferred if given the choice. I would probably have come to a very different conclusion, and I feel rather aggrieved and let down now. ID122

Forum users’ consent to hysteroscopy was shaped by comparisons to other gynaecological procedures, such as smear tests or intrauterine device (IUD) fittings. However, these descriptions often conflicted with actual experiences of hysteroscopy, challenging the validity of their consent:

I had one [hysteroscopy], they told me it was like a smear. It was not!!! [. . .] The whole thing was awful, but the worst part was that I was unprepared and thought it would be like a smear and was in no way expecting how invasive and long it would take. I fainted after it. ID671

With OPH framed as the only timely diagnostic option, consent to OPH was driven more by necessity and fear of symptom progression than by genuine preference:

The other reason why this is such a disgrace is that often women having this procedure have a reason to suspect cancer (as was my case) so you end up tolerating the pain through fear of waiting for months for the procedure to be done via GA [General Anaesthetic]. I put up with things that I definitely should not have done and would not have done in different circumstances. ID322

Forum users reported that severe pain, the risk of injury, and explicit pressure by clinicians often prevented them from withdrawing consent during the procedure:

A big problem is that you don’t know how painful it is until it starts. Then there is lots of implicit (and in my case explicit) pressure just to plough on and get it done ID161

Consent was often based on insufficient and inaccurate information. Patients faced an illusion of options limited to choosing between procedural pain and long GA wait times. Some felt unable to withdraw consent due to pain, clinician pressure, or fear of their symptoms worsening.

## Theme: unacknowledged vulnerability

Feelings of physical and emotional exposure and vulnerability were emphasised by forum users in their experiences. Physical exposure of the genital region induced feelings of vulnerability and defencelessness and highlighted a power-imbalance between clinician and patient:

I’d been waiting so long to get this sorted and having to be so exposed. I had a chair with those leg-rest things and I know they help the Doctor by being in the best position, but they make me feel like I am vulnerable. ID24

These vulnerabilities experienced throughout the procedure were compared to sexual violence:

I feel like I've been totally abused and violated. It’s been barbaric and medieval. [. . .] Where do I get support for the trauma this incident has caused me? I feel quite literally far, far worse than having been raped. ID32

History of sexual abuse or previous adverse gynaecological examinations heightened feelings of vulnerability:

I’d also told him that I was very uncomfortable with the procedure going ahead as I was still under psychology for sex abuse and would rather have gone home and prepared for it. ID1643

Adverse experiences had a lasting, unacknowledged impact on help-seeking behaviour and post-procedural comorbidities, heightening clinical vulnerability.

I developed a very severe phobia of all things medical last year, after a really bad experience with a Rapid Access clinic and hysteroscopy. I’ve been on anti-anxiety medication and Betablockers since. I started counselling 6 weeks ago. Unfortunately, it’s not getting better. I can’t go into a hospital or medical place. I don’t have any contact with any HCPs. [. . .] This literally will be the death of me, but I can’t get myself to go. ID3

Unacknowledged vulnerability during hysteroscopy was often marked by procedural exposure and exacerbated by pre-existing trauma. Adverse hysteroscopy experiences were compared to sexual abuse and linked to lasting psychological distress and avoidance of medical care.

## Theme: pain roulette

The analgesic protocol for hysteroscopy is unstandardised and can include GA or only non-steroidal anti-inflammatory drugs or paracetamol.^23^ There are inconsistencies in whether a patient receives GA or over-the-counter (OTC) pain relief for their procedure, and there is varied application of pain management policies between trusts and hospitals:

In the Board that I work for they offer spinal as an alternative to GA for hysteroscopy - in the Board I’ll be having it in, they don’t. It’s awake [with no pain medication] or GA (if you push for a GA or they decide you need it). They are adjoining Boards. The lack of consistency is baffling. ID4

This lack of equitable pain management informed an understanding that pain relief options were down to luck, which provoked a sense of injustice:

It [hysteroscopy pain relief] shouldn’t be the lottery that it is and it’s not fair that some women are allowed to be in agony and left traumatised. We should all get the same [options]. ID1355

Forum users disapproved of the forced binary choice for or against pain management during hysteroscopy procedures, when there is more choice given for other outpatient procedures:

There is nothing at all offered as an alternative; it’s either GA or nothing. No sedation or gas and air. I think it’s a bit daft. And quite a big waste of time. I have had a large hernia repair, a colonoscopy and an endoscopy, all done under sedation, so it doesn’t make sense – why not this. ID63

In some cases, the experience of analgesic roulette was experienced as a form of experimentation:

I feel like they chose me on purpose to experiment on to see if it was possible, if they could get away with it, or if they can do it to others. Then they blame my disability and mental health for me complaining that I've been severely harmed. I’m so angry. ID32

Forum users did recognise the difficulty of standardising pain relief when the pain experience could vary significantly:

Obviously this is something that is experienced very differently by different women [. . .] so I can see why this is a difficult thing to standardise. ID849

The lack of standardised protocols for hysteroscopy creates a system where access to adequate pain relief is inconsistent and arbitrary, leaving patients to navigate a “lottery” of care, where the reliance on trial-and-error analgesia was experienced as a form of medical disregard.

## Theme: gynaecological pain gaslighting

Medical gaslighting refers to the dismissal or minimisation of a patient’s symptoms, concerns, or experiences, including being blamed by healthcare professionals.^62,63^ Forum users described instances in which clinicians attributed painful hysteroscopy experiences to personal failings by the patient:

I was screaming and crying with the pain. The doctor was awful. Told me it was my fault it was taking so long and causing pain because I was overweight. ID302

Healthcare providers questioned the legitimacy of women’s pain experiences:

And then the doctor kept telling me that it shouldn’t hurt that much as if I was making up how much pain I was in. Absolutely awful [. . .]. It wasn’t far off feeling like I’d been tortured and then told off because it hurt. ID293

Medical records that contradicted patients’ lived pain experiences showed a disconnect between patient and clinician experience, and were seen to perpetuate this problem of misinformation at the outset of the hysteroscopy pathway:

I made it clear that I was in a lot of pain. The annoying part is my notes say ‘tolerated the procedure well’. Lies like that are why the literature falsely says it’s not that bad. ID655

Within the pre-procedural information while waiting for the intervention, hysteroscopy was positioned as “tolerable for most”, a perspective that was felt to be dishonest:

It’s clear that it is an intolerable procedure for some, and clinicians should be honest about that rather than gaslighting us with that “most people tolerate it” gaslighting crap. ID80

Forum users linked post-procedural co-morbidities to medical gaslighting and withholding of analgesia:

I was told before they started the hysteroscopy, I could have gas and air if I struggled, only to then be told “it’ll be over soon” and “it can’t be that painful” when I asked for it due to being in excruciating pain. 4 years later and I can still remember the pain and have vaginismus which started following those procedures. [. . .] I am now having pelvic physio and have been referred for psycho-sexual counselling, which could have been avoided had proper pain relief options been offered and had I (like many others) not been gaslit by health professionals. ID352

Accounts of medical gaslighting, where adverse experiences were dismissed, misrepresented in medical descriptions and records, or attributed to personal failings, are likely to impact long-term healthcare interactions.

## Theme: gendered pain gap

The data illustrated a profound sense of injustice, rooted in perceived gender biases in medical care: Posters traced this disparity to historic assumptions about female pain:

The expectation that childbirth is naturally painful therefore all gynae[cological] procedures should have the expectation of pain is archaic and horrendous. ID1188

This historical framing was felt to continue to shape and compromise modern clinical decision-making pointing to an inequality in expectations of pain-tolerance between male and female patients:

It’s nothing less than utter misogyny. A man would NEVER be expected to undergo such an invasive procedure with nothing. ID22

The normalisation of female pain through insufficient analgesic protocols in gynaecological procedures was frequently contested. The use of distraction techniques instead of effective pain relief represented the unequal consideration of female pain:

The idea that it’s ok to subject women to severe gynaecological pain as long as you chat to them about holidays, like a hairdresser, is abhorrent. ID97

The minimisation of female pain and the imposition of stereotypes that frame them as childlike or emotionally fragile are experienced as infantilising women.

I’ll tell you what I hate just as much [as insufficient pain relief] though is that patronising “good girl” when you’ve come down from the ceiling/stopped swearing/mashing the nurse’s hand. I’m not a [expletive] 3-year-old about to be given a lollipop, I’m a grown, middle-aged woman. ID608

Amid these inequities of treatment and pain-relief offering, forum users shared a prevailing sense of distrust explaining that self-advocacy became critical:

You need to really fight for yourself because medical practices don’t fight for women, we’re left to suffer. ID736

This proactive stance was framed as vital when navigating medical systems that marginalise women’s experiences. There is also a fundamental tension between the practical necessity of self-advocacy and the ethical injustice of requiring women to fight for appropriate care:

Women shouldn't have to “make a fuss” to get adequate pain relief. ID22

Forum users expressed a deep sense of discrimination, where gender biases in medical care normalise female pain, infantilise women, and create unequal standards for pain management, leading to a systemic lack of trust in healthcare providers and a prejudicial burden of self-advocacy.

## Discussion

### Main findings

The study is the first to qualitatively investigate online accounts of women’s experiences along the hysteroscopy pathway. It highlights that inadequate or misleading information compromises patients’ cognitive, emotional, and physiological preparation for the procedure undermining the validity of their consent. Emotional uncertainty, fear of harm, and physical exposure during hysteroscopy intersect to foster a problematic sense of helplessness and vulnerability. Disparities in clinical protocols resulted in considerable variability in pain management and an unpredictable patient experience. Adverse experiences were frequently perceived as being dismissed or invalidated by healthcare professionals, with some characterising this response as gaslighting. The perceived normalisation of women’s pain, conveying misogyny in healthcare practices heightened the need for forum users to self-advocate strongly in such situations of clinical vulnerability.

### Interpretation in the light of other evidence

Findings identify the hysteroscopy consent process as being insufficient and misleading, echoing data from other gynaecological procedures such as IUD insertions, cervical and pelvic examinations, where patients report unexpected pain, inadequate information, and the minimisation of discomfort.^28,64^ Symptom urgency and clinician pressure often compelled women to endure more pain than they would have otherwise chosen, which parallels findings from cervical cancer screening where well-intended encouragement can manifest as coercive practices.^65^ The adoption of validated, standardised consent checklists across NHS trusts could support shared decision-making and enhance psychological capability by ensuring that risks, alternatives, and pain expectations are consistently communicated. Forum users described how clinical set-up, genital exposure, and adverse clinician interactions contributed to feelings of vulnerability. Similar patterns are observed across routine gynaecological care,^66–68^ where vulnerability, embarrassment, and dehumanisation are commonly reported occurrences.

Unstandardised or inadequate pain management,^69,70^ combined with unknown pathological and psychological risk factors for OPH pain,^71^ create a “pain roulette” for patients reflected by varied hysteroscopy experiences. These diverse experiences suggest that current analgesic guidelines are insufficiently followed.^23,68,72^ Crucially, a stringent evaluation of their implementation could provide the foundation for policy reforms that standardise these guidelines across gynaecological care. These policies could reduce patient harm^73–76^ and reduce the strain on the healthcare system by decreasing short-term complications,^77^ unscheduled healthcare visits,^77^ and long-term healthcare avoidance.^78^

Forum users described their hysteroscopy experiences being trivialised, which supports findings from a parliamentary inquiry and extensive research on gynaecological procedures, which highlight the widespread dismissal of women’s symptoms.^28,28,64,79,80^ The long-term effects of this medical gaslighting include anxiety, depression, PTSD symptoms, trauma, distrust in healthcare providers, and avoidance of necessary medical treatment.^81–83^ Routine collection of post-procedure patient-reported outcome measures (PROMs) relating to pain, distress, and perceived trauma would support 360-degree feedback loops^84^ at both individual and service levels, supporting reflective practice and service improvement through the accountability of NHS trusts.

Hysteroscopy experiences were seen to represent a gendered pain bias, corroborating findings from other gynaecological domains.^29,80^ Findings suggest that the healthcare system fails to take women’s health seriously, forcing them to engage in high-level, pressurised self-advocacy. Forum users referred to their gendered experience as misogynistic and medical gaslighting, a term increasingly used in research investigating women’s healthcare experiences.^80,85,85–92^ To optimise patient communication and address gender bias, clinical training should include awareness of heuristics – practical, experience-based “rules of thumb” shaping judgement under uncertainty – as well as trauma-informed pelvic care. Critically, organisational factors including communication, clinician practice, and access to analgesia can support more positive patient experiences.^93^ As such, future qualitative research conducted within NHS trusts could usefully explore variation in practice and the conditions under which OPH is experienced positively.

Acknowledging and addressing patients’ negative healthcare experiences allows for an evaluation of current practices and improving the quality of women’s health care as set out by the UK’s Women’s Health Strategy.^94^ This study reveals that inconsistent analgesic protocols, opaque consent processes, and unacknowledged distress reflect broader structural neglect in women’s health care. This study highlights implications for clinical practice and the need for RCOG’s guidelines on informed decision-making, pain relief, and standardised consent for OPH,^23,95–97^ to be implemented as robust policies across NHS trusts.

### Limitations

A key strength of this study is its alignment with RCOG’s call for qualitative research into OPH experiences,^23^ providing valuable insights into organisational, clinical, and non-clinical factors while contextualising existing quantitative data on pain and satisfaction. Although demographic details of Mumsnet users are kept anonymised on the public forum, the current dataset included over 4700 diverse accounts from across the United Kingdom. The data featured organic, unprompted discussions of individuals with mental health conditions, special educational needs, comorbidities, and varying menopausal statuses, referral symptoms, and pregnancy statuses, providing a wide array of experiences.

While online data may not be fully representative of all hysteroscopy self-reporting, prior research supports the validity of online qualitative data in investigating gynaecological experiences.^28,29,80^ However, the potential influence of negativity bias must be acknowledged, as studies suggest that negative health experiences are more frequently shared online^93^ and may drive user engagement.^98^ Indeed, evidence that approximately 70%–80% of women tolerate the procedure in some settings^17,76,99^ highlights that negative experiences are not universal, nor should providers be uniformly characterised as dismissive or uncaring. Despite this, the data were representative of a spectrum of experiences as reflected by the themes, which align with offline mixed-methods research into OPH experiences,^21^ reinforcing their relevance to clinical practice.

## Conclusion

This study’s findings align with international recognition of systemic failings as outlined in the European Parliament’s definition of *gynaecological violence*. This includes procedures conducted without informed consent, using physical restraint, neglecting privacy and confidentiality, denial of pain relief, and the use of sexist, infantilising, or humiliating language. Such behaviours, though not necessarily intentional, represent broader structural issues, such as underfunded services, inadequate consent specific training, and entrenched power imbalances between clinicians and patients. This study positions hysteroscopy as a litmus test for examining the healthcare system’s engagement with women’s pain and medical autonomy, exposing the need for systemic reform in gynaecological practice. The prevention of post-procedural perceptions of gynaecological violence requires more than individual accountability: It demands institutional change and adoption of gold-standard consent processes within OPH.

## Supplemental Material

sj-pdf-1-whe-10.1177_17455057261440884 – Supplemental material for From pain gaslighting to gender biases in women’s accounts of hysteroscopy: A qualitative reflexive thematic analysis Supplemental material, sj-pdf-1-whe-10.1177_17455057261440884 for From pain gaslighting to gender biases in women’s accounts of hysteroscopy: A qualitative reflexive thematic analysis by Susanne K. Cromme, Richard Harrison and Katherine A. Finlay in Women's Health
